# Clinical and Subclinical Atherosclerotic Disease in Adolescents With Familial Hypercholesterolemia

**DOI:** 10.1016/j.jaccas.2025.106600

**Published:** 2026-01-21

**Authors:** Sura Kishore Mishra, Pankaj Kumar Maravi, Bitan Mohanty, Santoshi Shamad

**Affiliations:** a Department of Cardiology, S.C.B Medical College, Cuttack, Odisha, India; b S.C.B Medical College, Cuttack, Odisha, India

**Keywords:** coronary artery disease, drug-coated balloon (DCB), drug-eluting stent (DES), familial hypercholesterolemia (FH), plasma exchange (PLEX)

## Abstract

Familial hypercholesterolemia is primarily a disorder of reduced low-density lipoprotein (LDL) clearance, which can be inherited in either homozygous or heterozygous form. Despite the availability of various modalities to achieve target LDL cholesterol levels, inadequate control remains a significant risk factor for accelerated atherosclerosis in the pediatric population. Moreover, the occurrence of coronary artery disease in adolescents poses unique management challenges given the limited representation of this age group in clinical trials. We report 2 cases of coronary artery disease and 1 case of subclinical atherosclerosis in adolescent patients with genetically confirmed familial hypercholesterolemia, outlining the diagnostic work-up, challenges in achieving LDL cholesterol control, measures undertaken, including plasma exchange, and revascularization strategies using drug-eluting stents or drug-coated balloons, in accordance with current guidelines.

Familial hypercholesterolemia (FH) is the most common genetic disorder of lipid metabolism, with a worldwide prevalence of 1 in 300 for the heterozygous form and 1 in 400,000 for the homozygous form.^1^ FH is caused by inherited autosomal-dominant defects of low-density lipoprotein (LDL) metabolism. There are 3 major genetic loci related to FH, with the majority of cases due to mutations in the LDL receptor gene. The pathophysiologic basis of FH is accelerated atherosclerosis due to the cumulative exposure to high concentrations of LDL. Although the prevalence of FH in the general population is substantial, only about 15% to 20% of affected patients receive a formal diagnosis.^2^ Patients with untreated heterozygous FH have an approximately 10- to 20-fold increased risk of premature coronary artery disease (CAD).^2^^,^^3^ This risk can be reduced to that of the general population with appropriate recognition and treatment of FH.^2^^,^^4^, ^5^, ^6^

## Case 1

A 17-year-old young woman presented to the emergency department with chest pain at rest. She had experienced exertional angina (NYHA functional class II) for the previous 3 months before presentation, which had progressed to class III over the preceding 2 days. She had previously been diagnosed with dyslipidemia and had been on standard lipid-lowering therapy for the past 3 months, with no reported family history of premature CAD.

**Figure 1:**
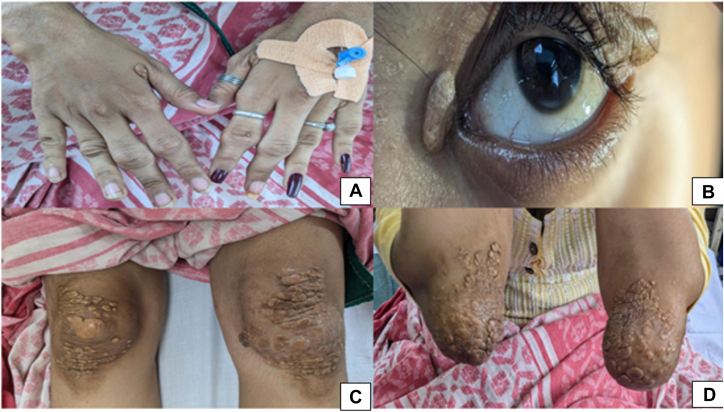
Cutaneous Manifestations of FH in Case 1 (A, C, and D) Tuberoeruptive xanthomas. (B) Xanthelasma and arcus cornealis. FH = familial hypercholesterolemia.

Her vital signs were stable. Examination revealed multiple tuberoeruptive xanthomas over the extensor aspects of the proximal interphalangeal joints, bilateral knees, and elbows, along with bilateral xanthelasma and arcus cornealis (Figure 1).

**Figure 2:**
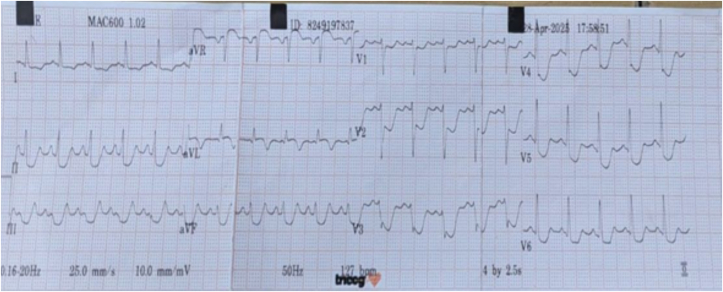
Electrocardiogram of Case 1 Twelve-lead electrocardiogram showing significant ST-segment elevation in lead aVR and marked ST-segment depression in leads II, III, aVF, and V_1_ to V_6_.

**Figure 3:**
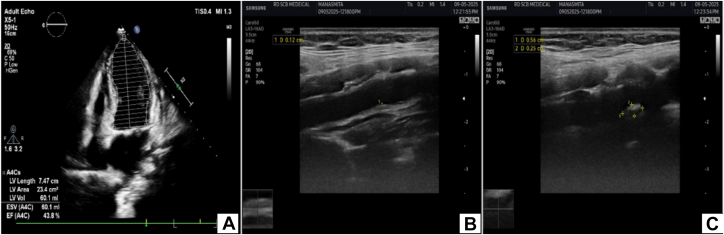
Carotid Intima-Media Thickness of Case 1 (A) Echocardiography showing an ejection fraction of 42%. (B) Ultrasound Doppler showing increased CIMT in the right common carotid artery. (C) Ultrasound Doppler showing calcified plaque of 5 × 3 mm at the bulb of the left common carotid artery. CIMT = carotid intima-media thickness.

Electrocardiography demonstrated ST-segment depression in the precordial and limb leads and ST-segment elevation in lead aVR (Figure 2). Cardiac troponin was elevated. Transthoracic echocardiography revealed regional wall motion abnormalities in the left anterior descending artery (LAD) territory, a left ventricular ejection fraction of 44% (Figure 3A), and minimal pericardial effusion. The patient's GRACE and TIMI risk scores were 74 and 5, respectively.

Laboratory investigations showed hemoglobin: 10.5 g/dL, hemoglobin A1c: 5.2%, total cholesterol: 538 mg/dL, triglycerides: 67 mg/dL, high-density lipoprotein cholesterol: 38 mg/dL, LDL cholesterol (LDL-C): 486 mg/dL, and lipoprotein (a): 156.2 mg/dL. Her Dutch Lipid Clinic Network score for FH was >8, indicating a diagnosis of definite FH.

**Figure 4:**
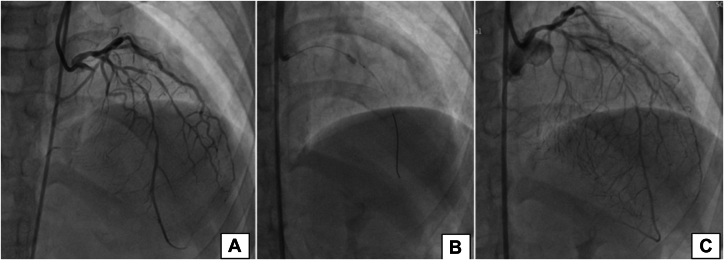
Coronary Angiography and Percutaneous Coronary Intervention of Case 1 (A) Right anterior oblique cranial view showing mid to distal LAD stenosis (80%-90%) with the tightest portion across the D2. (B) Wire in the LAD and balloon in the mid LAD across the D2. (C) Results after deployment of drug-coated balloon. D2 = second diagonal branch; LAD = left anterior descending artery.

Coronary angiography revealed 30% stenosis in the distal left main coronary artery (LMCA), mid-to-distal LAD diffusely diseased with the tightest portion of 10 mm across the second diagonal branch having 95% stenosis (Figure 4A), left circumflex artery across the second obtuse marginal branch 80% × 10 mm (Medina 1,1,1), distal left circumflex artery 80% × 6 mm, ostioproximal second obtuse marginal branch 60% × 14 mm, right coronary artery ostial 50% to 60%, and diffuse proximal-to-distal right coronary artery disease 60% to 80%, with a SYNTAX score of 26. Carotid Doppler demonstrated increased carotid intima-media thickness (CIMT) bilaterally and a calcified plaque (5 × 3 mm) at the left common carotid artery (CCA) bulb (Figures 3B and 3C).

Genetic analysis confirmed heterozygous FH, and the patient was confirmed to carry a pathogenic variant in the *LDLR* gene (ENST500000558518.6) located in exon 3, according to American College of Medical Genetics and Genomics standards. Cascade screening identified both parents as carriers. Given persistently elevated LDL-C despite maximal therapy and unavailability of LDL apheresis, she underwent 5 sessions of plasma exchange, reducing her LDL-C to 158 mg/dL and lipoprotein (a) to 29.5 mg/dL (Table 1). She subsequently underwent drug-coated balloon (DCB) angioplasty to the LAD, achieving improved myocardial perfusion and symptomatic relief.

**Table 1:** Lipid Profiles at Baseline and After Each Plasma Exchange Session

Lipid Profile	First Visit	Plasma Exchange				
		After Session 1	After Session 2	After Session 3	After Session 4	After Session 5
TC	483	218	177	220	183	200
TG	62	56	64	99	67	83
HDL-C	33	31	33.9	34	33	35
LDL-C	>380	169	142	149	145	158
VLDL-C	12.4	11	12.8	20	13.4	16.6
ApoA1	79	87	89	—	74	82
Lp(a)	156.2	76	75.8	—	24.6	29.5

ApoA1 = apolipoprotein A1; HDL-C = high-density lipoprotein cholesterol; LDL-C = low-density lipoprotein cholesterol; Lp(a) = lipoprotein (a); TC = total cholesterol; TG = triglycerides; VLDL-C = very-low-density lipoprotein cholesterol.

### Intervention

The LMCA was cannulated using a 6-F guide. The LAD lesion was crossed with a Runthrough intermediate guidewire. Sequential predilation was performed across the second diagonal branch using a 1.25 × 10 mm SC balloon, a 1.5 × 10 mm SC balloon, and a 2.5 × 10 mm NC balloon for optimal lesion preparation. A DCB (paclitaxel) Prevail balloon (3.0 × 10 mm) was deployed at 8 atm for 60 seconds in the mid LAD across the second diagonal branch (Figure 4B), and final vessel diameter was 2.99 with TIMI flow grade 3 (Figure 4C).

### Follow-Up

The patient was referred to another center for PCSK9 inhibitor therapy and was symptom free at 3 months.

## Case 2

A 14-year-old young man with a diagnosed case of FH for 3 years on statin therapy presented to the emergency department with reports of chest pain at night awakening him from sleep for the past 10 to 12 days. There was no family history of premature CAD. His vital signs were stable. He had multiple xanthomas over the extensor aspects of the knees and elbows.

Electrocardiogram showed ST-segment elevation in aVR and V1 to V3 with poor R-wave progression and reciprocal ST-segment depression in leads II, III, aVF, and V5-V6. Echocardiography showed global hypokinesia of the left ventricle with trivial mitral regurgitation and mild left ventricular systolic dysfunction. Baseline LDL-C was 361 mg/dL, with other biochemical markers within normal limits.

**Figure 5:**
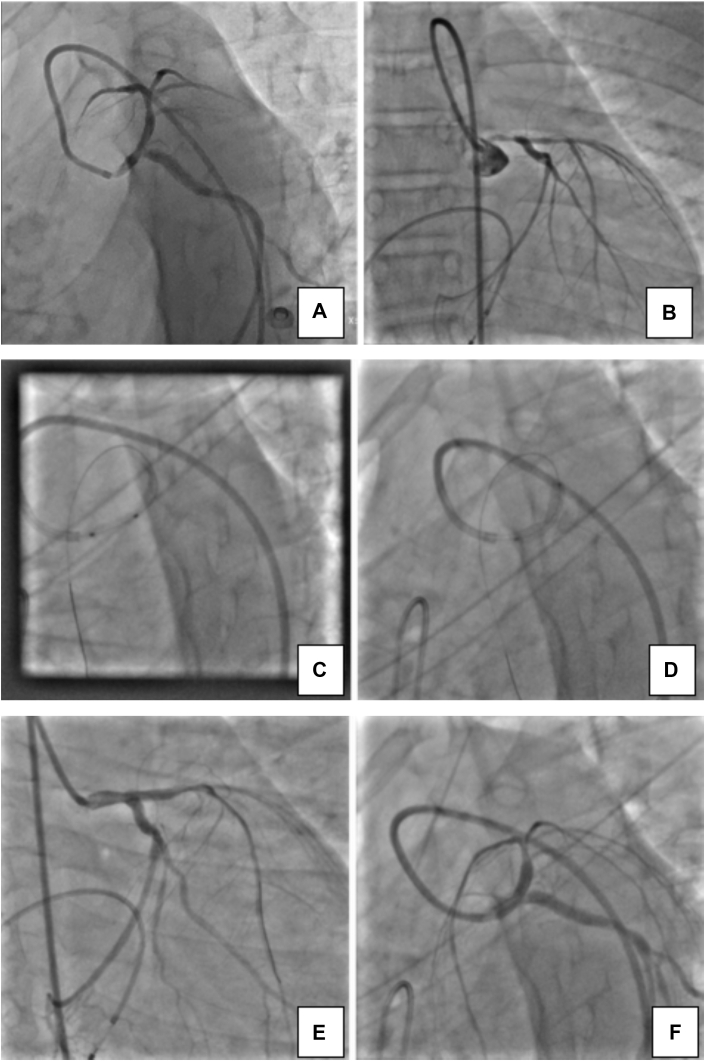
Coronary Angiography and Percutaneous Coronary Intervention of Case 2 (A) Coronary angiography left anterior oblique caudal view showing ostial LMCA 99%, distal LMCA 80%, and (B) mid LAD 70% lesions. (C) DES implantation in the proximal LMCA. (D) DES implantation from the LMCA to LAD. (E and F) postprocedural final result. DES = drug-eluting stent; LAD = left anterior descending artery; LMCA = left main coronary artery.

Coronary angiography showed ostial LMCA 99%, distal LMCA 80%, and mid LAD 70% lesions with minor plaque in the ostial left circumflex artery and nondominant right coronary artery (Figures 5A and 5B). As the patient was a minor, consent was sought from the parents, who did not agree to a surgical procedure. Percutaneous transluminal coronary angioplasty with drug-eluting stent (DES) to the LMCA and the LMCA-to-LAD was performed (Figures 5C and 5D). Intravascular ultrasound showed good apposition of the stent. Images are currently not available for reference, as an outsourced machine was used during the procedure. The patient was confirmed to have a homozygous pathogenic variant in the *LDLR* gene (ENST00000558518.1) located in intron 7, as per American College of Medical Genetics and Genomics standards.

### Intervention

The lesion in the LMCA was crossed with a Runthrough floppy guidewire and was predilated with 2.0 × 10 mm Across HP balloon and 2.5 × 12 mm NC Traveler balloon, then stenting was performed with a 4.0 × 9 mm Resolute Integrity stent deployed at 14 atm pressure. Postdilatation was performed with a 4.0 × 8 mm NC Traveler balloon. The distal LMCA-to-LAD lesion was crossed with the same wire, and a 3.5 × 22 mm Resolute Integrity stent was deployed at 14 atm pressure. Postdilatation was performed with a 4.0 × 8 mm NC Traveler balloon. Distal TIMI flow grade 3 was achieved (Figures 5E and 5F).

### Follow-Up

The patient was discharged on guideline-directed medical therapy. One month later, exertion-related chest pain emerged, progressing from NYHA functional class II to III over 2 months despite medications. Computed tomography angiography indicated 70% in-stent restenosis in the LMCA and 80% in the LMCA-to-LAD stents. DCB was considered before CABG, but the appropriate-sized hardware was not available. However, the patient succumbed during CABG surgery.

## Case 3

**Figure 6:**
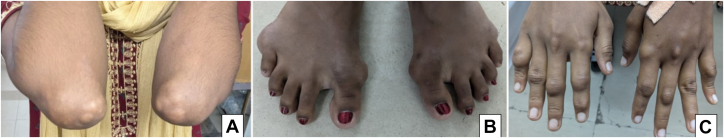
Cutaneous Manifestations of FH in Case 3 (A to C) Tuberoeruptive xanthomas. FH = familial hypercholesterolemia.

A 13-year-old young woman with a history of uncontrolled dyslipidemia on lipid-lowering therapy presented for further evaluation. There was no family history of premature CAD. Her vital signs were stable. Multiple tuberoeruptive xanthomas were observed over the proximal interphalangeal, metacarpophalangeal, and metatarsophalangeal joints, as well as the elbows (Figure 6). Electrocardiogram was normal, and echocardiography showed an ejection fraction of 68%. Laboratory results indicated hemoglobin: 10.8 g/dL and hemoglobin A1c: 4.9%, and lipid profile indicated cholesterol: 516 mg/dL, triglycerides: 161 mg/dL, high-density lipoprotein cholesterol: 21 mg/dL, and LDL-C: 437 mg/dL. Her Dutch Lipid Clinic Network score of >8 indicated definite FH.

Carotid Doppler revealed increased CIMT (left: 0.8 mm, right: 0.4 mm) with calcified plaques (right CCA anterior: 3.5 cm, right CCA posterior: 1.8 cm, left CCA anterior: 2.8 cm) without significant stenosis. Her treadmill test was negative for provocable ischemia at 10 metabolic equivalents and achieved 100% maximum predicted heart rate. Genetic analysis confirmed heterozygous FH due to an LDL receptor mutation (*LDLR* gene, ENST500000558518.6, exon 4). The patient was receiving rosuvastatin 40 mg and ezetimibe 10 mg once daily; however, lipid levels remained uncontrolled. She was therefore referred to another center for initiation of PCSK9 inhibitor therapy.

## Discussion

FH remains primarily a clinical diagnosis, although genetic testing can confirm pathogenic variants. North American and European diagnostic criteria from U.S. MEDPED (Make Early Diagnosis to Prevent Early Death),^7^ Simon Broome,^8^ and the Dutch Lipid Clinic Network^9^ (Table 2) integrate family history, physical findings, LDL-C levels, and genetic testing, most often involving mutations in the *LDLR*, *APOB*, and *PCSK9* genes. The International FH Foundation recommends genetic testing in patients with probable or definite FH, with cascade screening of first-degree relatives.^10^ In our 3 patients, FH was diagnosed clinically and was subsequently confirmed with genetic testing for gene locus and zygosity.^10^

**Table 2:** U.S. MEDPED, Simon Broome, and Dutch Lipid Clinic Network Criteria for the Diagnosis of FH

U.S. MEDPED Criteria^7^				
Age (y)	First-Degree Relative With FH	Second-Degree Relative With FH	Third-Degree Relative With FH	General Population
<20	220 mg/dL	230 mg/dL	240 mg/dL	270 mg/dL
20-29	240 mg/dL	250 mg/dL	260 mg/dL	290 mg/dL
30-39	270 mg/dL	280 mg/dL	290 mg/dL	340 mg/dL
≥40	290 mg/dL	300 mg/dL	310 mg/dL	360 mg/dL
Simon Broome Criteria^8^				
Criteria				Possibility
In adults: TC >7.5 mmol/L (290.0 mg/dL) (or when available, LDL-C >4.9 mmol/L [189.5 mg/dL]) In pediatric patients: TC >6.7 mmol/L (259.1 mg/dL), or LDL-C >4.0 mmol/L (154.7 mg/dL), *AND*				Definite
Tendon xanthoma in the patient or first-/second-degree relative, *OR*				
Presence of *LDLR*, *APOB*, or *PCSK9* gene mutation				
In adults: TC >7.5 mmol/L (290.0 mg/dL) (or when available, LDL-C >4.9 mmol/L [189.5 mg/dL]) In pediatric patients: TC >6.7 mmol/L (259.1 mg/dL), or LDL-C >4.0 mmol/L (154.7 mg/dL), *AND*				Possible
Family history of MI <50 years old in second-degree relative or <60 years old in first-degree relative *OR* alternatively				
Family history of TC >7.5 mmol/L (290.0 mg/dL) in a first- or second-degree relative				
Dutch Lipid Clinic Network Criteria^9^				
Category				Score
Family history				
Premature CVD (men <55 y, women <60 y) in first-degree relative, *OR*				1
LDL >95th percentile in first-degree relative *AND/OR*				1
Tendon xanthoma and/or arcus cornealis in first-degree relative, *OR*				2
LDL >95th percentile in children <18 years old				2
Personal history				
Premature CAD in patient (men <55 y, women <60 y)				2
Premature cerebral or peripheral vascular disease (men <55 y, women <60 y)				1
Clinical examination				
Tendon xanthomas, *OR*				6
Corneal arcus younger than 45 y				4
LDL-C				
>330 mg/dL (8.5 mmol/L)				8
250-329 mg/dL (6.5-8.5 mmol/L)				5
190-249 mg/dL (4.9-6.4 mmol/L)				3
155-189 mg/dL (4.0-4.9 mmol/L)				1
Presence of functional LDL receptor mutation (in *LDLR*, *APOB*, or *PCSK9* gene)				8
Diagnosis based on overall score				
Definite				>8
Probable				6-8
Possible				3-5
Unlikely				<3

CAD = coronary artery disease; CVD = cardiovascular disease; FH = familial hypercholesterolemia; LDL = low-density lipoprotein; LDL-C = low-density lipoprotein cholesterol; MEDPED = Make Early Diagnosis to Prevent Early Death; MI = myocardial infarction; TC = total cholesterol.

Refining risk stratification with measures of subclinical coronary atherosclerosis in asymptomatic FH individuals appears promising. However, within the context of FH, atherosclerosis imaging identifies individuals eligible for aggressive management. Modalities studied include CIMT, coronary artery calcium scoring, and CCTA. Unlike our first 2 cases with confirmed CAD, case 3 involved an asymptomatic FH patient with CIMT indicating subclinical atherosclerosis, necessitating stringent LDL-C control to prevent CAD progression.

Statins, ezetimibe, and PCSK9 inhibitors have improved FH prognosis, yet many patients fail to achieve target LDL-C levels despite maximal therapy. This underscores the need for novel LDL-lowering strategies independent of LDL receptor activity, particularly in homozygous FH, however limited availability and high costs restrict their routine use.^1^ Lipoprotein apheresis effectively lowers apoB-containing lipoproteins, including LDL-C and lipoprotein(a). Despite U.S. Food & Drug Administration (FDA) approval for high-risk patients with FH who are refractory to conventional therapy, lipoprotein apheresis remains underused.^11^ Selective lipoprotein apheresis is preferred, although plasmapheresis serves as an alternative.^12^

FDA-approved indications for apheresis are as follows^11^:

1. Clinically diagnosed homozygous FH with LDL-C >500 mg/dL
2. Clinically diagnosed heterozygous FH with LDL-C >300 mg/dL
3. Clinically diagnosed heterozygous FH with LDL-C >100 mg/dL with documented CAD/peripheral artery disease
4. Clinically diagnosed heterozygous FH with LDL-C >100 mg/dL with documented CAD/peripheral artery disease with lipoprotein(a) >60 mg/dL

Emerging therapeutics include mipomersen (mRNA inhibition) and lomitapide (VLDL [very low-density lipoprotein] inhibition), both FDA-approved for homozygous FH. Inclisiran, a small interfering RNA targeting *PCSK9*, is under investigation.^13^ In our first case, despite high-dose statins and ezetimibe, LDL-C >400 mg/dL prompted plasma exchange for short-term reduction prior to revascularization. In case 2, treatment was limited to high-dose statin and ezetimibe before revascularization. Case 3 followed a similar pattern, and the patient was referred for PCSK9 inhibitor therapy. LDL-C reduction in FH is associated with improved outcomes, including plaque regression and reduced cardiovascular events. Meeting LDL targets allows FH patients to approach population-level myocardial infarction risk. Lipid lowering is the cornerstone of primary and secondary prevention, but revascularization in FH-associated CAD is inadequately studied.^13^

In the non-FH population, percutaneous coronary intervention versus CABG comparisons show that CABG benefits patients with diabetes, triple-vessel disease, and high SYNTAX scores by addressing current and future lesions. This may apply to FH patients with accelerated atherosclerosis. However, CABG in young FH patients includes challenges such as early graft failure and reluctance to undergo a surgical procedure.^13^ In case 1, the patient's guardians declined surgery; in case 2, CABG after failed percutaneous coronary intervention led to periprocedural death.

DESs are generally preferred, but permanent scaffolds pose challenges in young FH patients with progressive disease. DESs underperform in diffuse disease with higher restenosis. DCBs deliver antiproliferative agents without scaffolds, suitable for diffuse lesions, small vessels, and FH, treating longer segments with minimal stent length.^13^^,^^14^ In case 1, DCB to the mid LAD achieved desirable perfusion and sustained symptom relief over 3 months. Conversely, DES implantation in case 2 led to in-stent restenosis, highlighting the limitations of a conventional strategy.

In summary, aggressive lipid lowering is fundamental in FH, with apheresis and novel agents beneficial when conventional therapy is insufficient. Revascularization requires careful consideration, paralleling diabetics in terms of burden and recurrence. DCBs show promise in selected cases, however further studies are needed to determine the optimal approach.

## Conclusions

FH is a chronic disorder that predisposes patients to premature and progressive CAD. Despite advances in lipid-lowering therapies, a substantial proportion of patients fail to achieve guideline-recommended LDL-C levels, necessitating adjunctive strategies such as lipoprotein apheresis and novel agents. Our cases highlight both the challenges and opportunities in managing FH-associated CAD, demonstrating that aggressive lipid lowering should precede and complement revascularization. Given the diffuse and progressive nature of atherosclerosis in FH, individualized revascularization strategies are crucial. While CABG may provide long-term benefit in selected high-burden patients, younger age and graft durability remain concerns. Similarly, newer modalities such as DCBs may offer advantages in diffuse disease where conventional stenting underperforms. Ultimately, integrating optimal medical therapy with carefully selected interventional approaches offers the best chance of improving outcomes in this high-risk population.

**Central Illustration:**
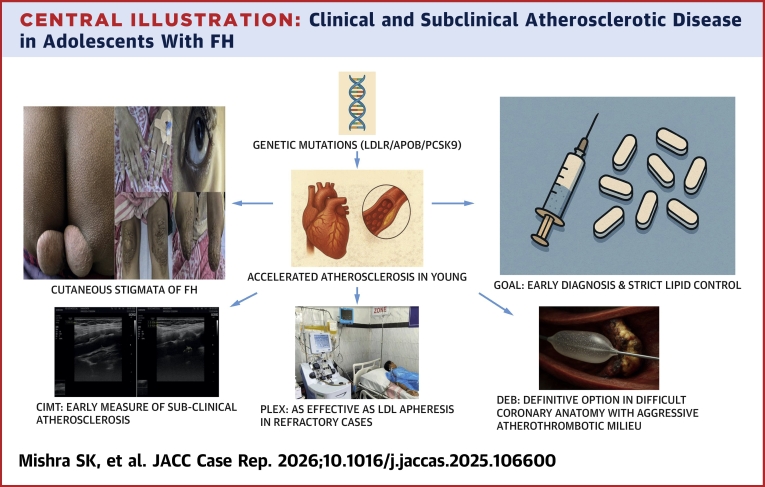
Clinical and Subclinical Atherosclerotic Disease in Adolescents With FH CIMT = carotid intima-media thickness; DEB (DCB) = drug-coated balloon; FH = familial hypercholesterolemia; PLEX = plasma exchange.

## Funding Support and Author Disclosures

The authors have reported that they have no relationships relevant to the contents of this paper to disclose.

**Take-Home Messages**

• Early FH diagnosis with cascade family screening and CIMT enables targeted lipid lowering therapy to prevent CAD.
• Plasma exchange is still a valid alternative to LDL apheresis in the current era in refractory cases.
• A patient-centered revascularization plan should be tailored, selecting percutaneous coronary intervention or coronary artery bypass grafting based on age, genotype, lesion complexity, and long-term outcomes to optimize efficacy and durability; drug-coated balloons can be a promising option alternative to drug-eluting stents in difficult coronary artery anatomy with aggressive atherothrombotic milieu.

**Equipment List:** 

Equipment list for case 1 (vessel addressed: mid LAD across D2; approach: right femoral artery)
Guide catheter: 6-F JL 3.5; guidewire: 0.014-inch × 180-cm Runthrough intermediate guidewire (Terumo)
Balloons: 1.25 × 10 mm SC balloon,1.5 × 10 mm SC balloon, 2.5 × 10 mm NC balloon
3.0 × 10 mm DCB Prevail balloon (Medtronic).
Equipment list for case 2 (vessels addressed: LMCA and LMCA to LAD; approach: right femoral artery)
Guide catheter: 7-F JR 3.5 and 7-F XBLAD catheter (Cordis) 3.5; guidewire: 0.014-inch × 180-cm Runthrough floppy guidewire (Terumo)
Balloons: 2.0 × 10 mm Across HP (Acrostak), 2.5 × 12 mm NC Traveler (Abbott), 4.0 × 8 mm NC Traveler (Abbott) balloons
Stents: 3.5 × 22 mm Resolute Integrity stent (LMCA to LAD) (Medtronic), 4.0 × 9 mm Resolute Integrity stent (Medtronic)

D2 = second diagonal branch; DCB = drug-coated balloon; LAD = left anterior descending artery; LMCA = left main coronary artery; NC = noncompliant; SC = semicompliant.

## References

[1] Choi D., Malick W.A., Koenig W., Rader D.J., Rosenson R.S. (2023). Familial hypercholesterolemia: challenges for a high-risk population JACC focus seminar 1/3. J Am Coll Cardiol.

[2] Bilen O., Pokharel Y., Ballantyne C.M. (2015). Genetic testing in hyperlipidemia. Cardiol Clin.

[3] Watts G.F., Lewis B., Sullivan D.R. (2007). Familial hypercholesterolemia: a missed opportunity in preventive medicine. Nat Clin Pract Cardiovasc Med.

[4] Gidding S.S., Champagne M.A., De Ferranti S.D. (2015). The agenda for familial hypercholesterolemia: a scientific statement from the American Heart Association. Circulation.

[5] Goldberg A.C., Hopkins P.N., Toth P.P. (2011). Familial hypercholesterolemia: screening, diagnosis and management of pediatric and adult patients: clinical guidance from the National Lipid Association Expert Panel on Familial Hypercholesterolemia. J Clin Lipidol.

[6] Santos R.D., Gidding S.S., Hegele R.A. (2016). Defining familial hypercholesterolemia and the implications for clinical management: a consensus statement from the International Atherosclerosis Society Severe Familial Hypercholesterolemia Panel. Lancet Diabetes Endocrinol.

[7] Williams R.R., Hunt S.C., Schumacher M.C. (1993). Diagnosing heterozygous familial hypercholesterolemia using new practical criteria validated by molecular genetics. Am J Cardiol.

[8] Scientific Steering Committee on behalf of the Simon Broome Register Group (1991). Risk of fatal coronary heart disease in familial hypercholesterolemia. BMJ.

[9] World Health Organization (1998). Familial Hypercholesterolemia (FH): Report of a second WHO consultation. http://whqlibdoc.who.int/hq/1999/WHO_HGN_FH_CONS_99.2.pdf.

[10] Sturm A.C., Knowles J.W., Gidding S.S. (2018). Clinical genetic testing for familial hypercholesterolemia: *JACC* Scientific Expert Panel. J Am Coll Cardiol.

[11] Gianos E., Duell P.B., Toth P.P. (2024). Lipoprotein apheresis: utility, outcomes, and implementation in clinical practice: a scientific statement from the American Heart Association. Arterioscler Thromb Vasc Biol.

[12] France M., Rees A., Datta D. (2016). HEART UK statement on the management of homozygous familial hypercholesterolaemia in the United Kingdom. Atherosclerosis.

[13] Ungar L., Sanders D., Becerra B., Barseghian A. (2018). Percutaneous coronary intervention in familial hypercholesterolemia is understudied. Front Cardiovasc Med.

[14] Korjian S., McCarthy K.J., Larnard E.A. (2024). Drug-coated balloons in the management of coronary artery disease. Circ Cardiovasc Interv.

